# Inadvertent insertion of a venous catheter into the internal thoracic vein: a word of caution

**DOI:** 10.1590/1677-5449.190097

**Published:** 2019-11-18

**Authors:** Flavia Ramos Tristão, Ricardo César Rocha Moreira, Carlos Eduardo Del Valle, Giana Caroline Strack Neves

**Affiliations:** 1 Hospital Nossa Senhora das Graças – HNSG, Serviço de Cirurgia Vascular Prof. Dr. Elias Abrão, Curitiba, PR, Brasil.; 2 Universidade Federal do Paraná – UFPR, Hospital de Clínicas, Serviço de Ultrassonografia Vascular com Doppler, Curitiba, PR, Brasil.

**Keywords:** catheters, adjuvant chemotherapy, vascular surgical procedures, cateteres, quimioterapia adjuvante, procedimentos cirúrgicos vasculares

## Abstract

Central venous catheters are widely used in clinical practice and are linked to many types of complications, including incorrect positioning at the time the catheter is fitted. Here, the authors describe a case in which a fully implantable catheter was inadvertently positioned in the right internal thoracic vein. The complication was identified when the nursing team attempted to use the catheter. The right internal thoracic vein is within the radiographic projection of the right brachiocephalic vein and the superior vena cava, simulating correct catheter placement on an anteroposterior radiograph. In cases of central catheter malfunction during the immediate postoperative period, work-up should include oblique and lateral views, to rule out the complication described here without a need for computed tomography.

## Resumo

Os cateteres venosos centrais são amplamente usados na prática clínica e estão relacionados a vários tipos de complicações, entre elas o mau posicionamento no momento do implante do cateter. Os autores relatam o caso de um cateter totalmente implantável que foi inadvertidamente posicionado na veia torácica interna direita, sendo a complicação reconhecida após tentativa do uso do cateter na enfermaria. A veia torácica interna direita fica na projeção da veia inominada direita e da veia cava superior, simulando um bom posicionamento do cateter na radioscopia em projeção anteroposterior. Em caso de mau funcionamento de um cateter central no pós-operatório imediato, a conduta deve incluir imagens oblíquas ou de perfil, para excluir a complicação relatada neste trabalho sem necessidade de tomografia computadorizada.

## INTRODUCTION

Central venous catheters are devices used when access to the central veins is needed for hyperosmolar drugs or chemotherapy, dialysis, blood products, parenteral nutrition, central venous pressure measurement, pulmonary artery catheterization, or cardiac pacing and for general venous access requirements in case of non-availability of peripheral access.^1^^,^ 2 The veins most commonly used for central catheter placement are the internal jugular veins and subclavian veins, followed by the femoral veins and superficial veins of the upper limb (for peripherally inserted central catheters). Central catheter placement must be performed with strict awareness of potential complications, which are usually divided into three categories: mechanical, infectious, and thrombotic. The three most common mechanical complications at the time of insertion are arterial puncture, hematoma, and pneumothorax.^2^^,^ 3 Other complications include hydrothorax, hemothorax, chylothorax, extravasation of infusate, cardiac tamponade, mediastinal hemorrhage, and malpositioning of the catheter.^1^^-^ 3 Catheter malpositioning can cause malfunction and can also lead to other complications, like jugular or subclavian retrograde infusion and the other complications mentioned above, e.g. mediastinal or pleural bleeding. The case reported in this paper is much less frequent, involving inadvertent insertion of the catheter into the right internal thoracic vein. This particular form of malpositioning can be difficult to detect in either supine fluoroscopy or supine chest radiographs, because these views show the internal thoracic vein superimposed over the superior vena cava. The objective of this paper, beyond reporting the clinical case, is to describe the pitfalls that can lead to failure to recognize this type of malposition and how to avoid them.

## CASE DESCRIPTION

**Figure 1:**
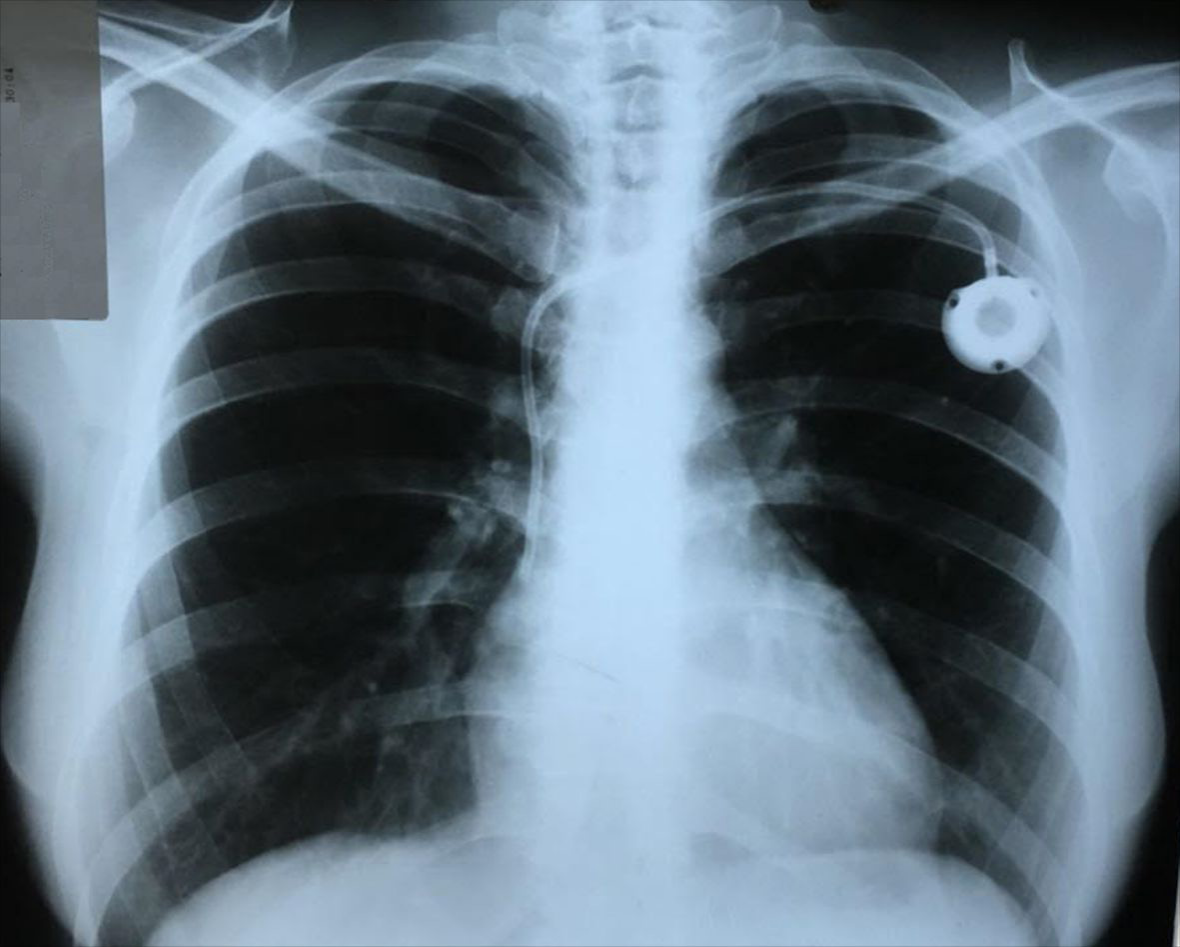
Anteroposterior chest radiograph taken in the immediate postoperative period, showing unremarkable positioning of the catheter.

A 49-year-old woman was admitted to our hospital for chemotherapy treatment of a right breast adenocarcinoma. She was scheduled for insertion of a long-stay central venous catheter. She had no previous history of central catheter insertion. On examination, no abnormalities of the neck or veins were found. A 9-French totally implanted catheter was inserted through the left subclavian vein under local anesthesia and sedation, under fluoroscopy, with adequate retrograde flow through the sheath and without any adverse events. Her anteroposterior chest radiograph showed the catheter in the desired position, without pneumothorax (Figure 1).

**Figure 2:**
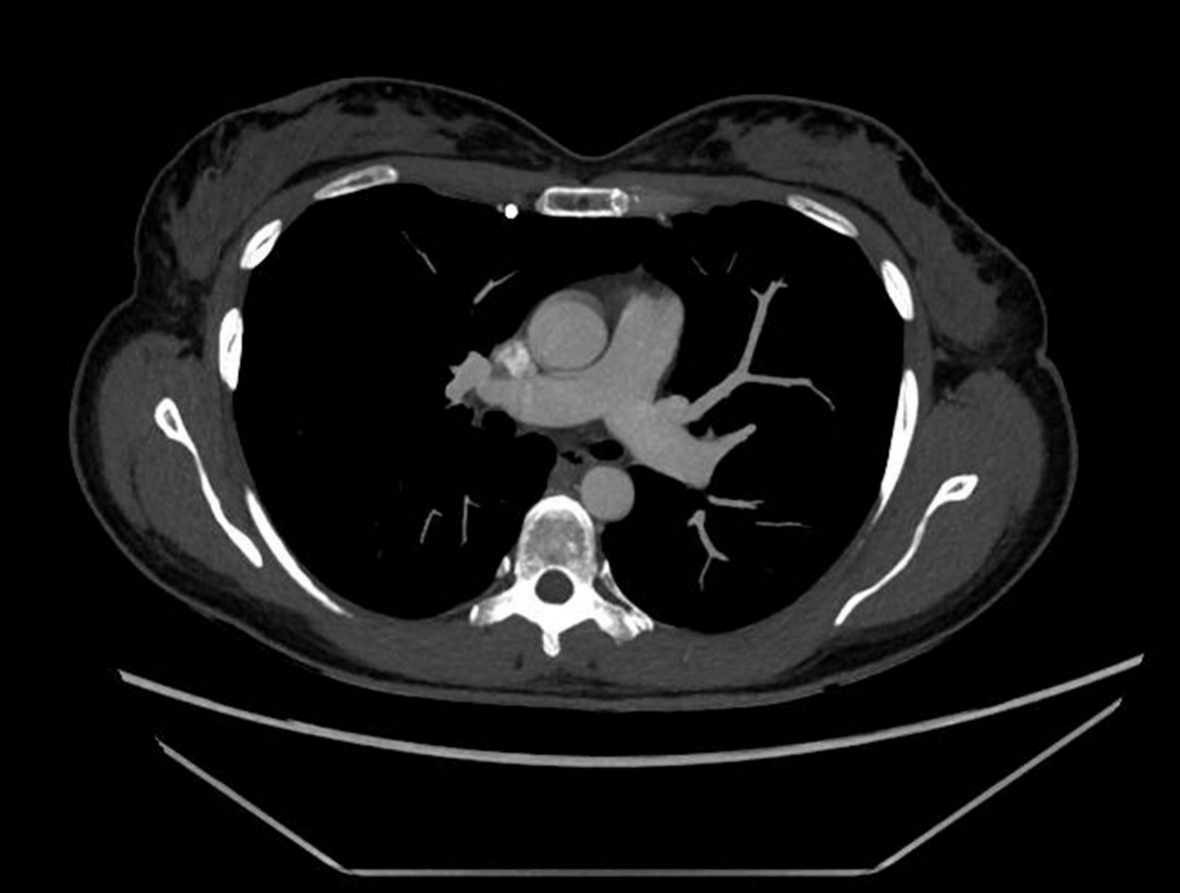
Axial computed tomography image showing the port-a-cath positioned behind the anterior chest wall.

**Figure 3:**
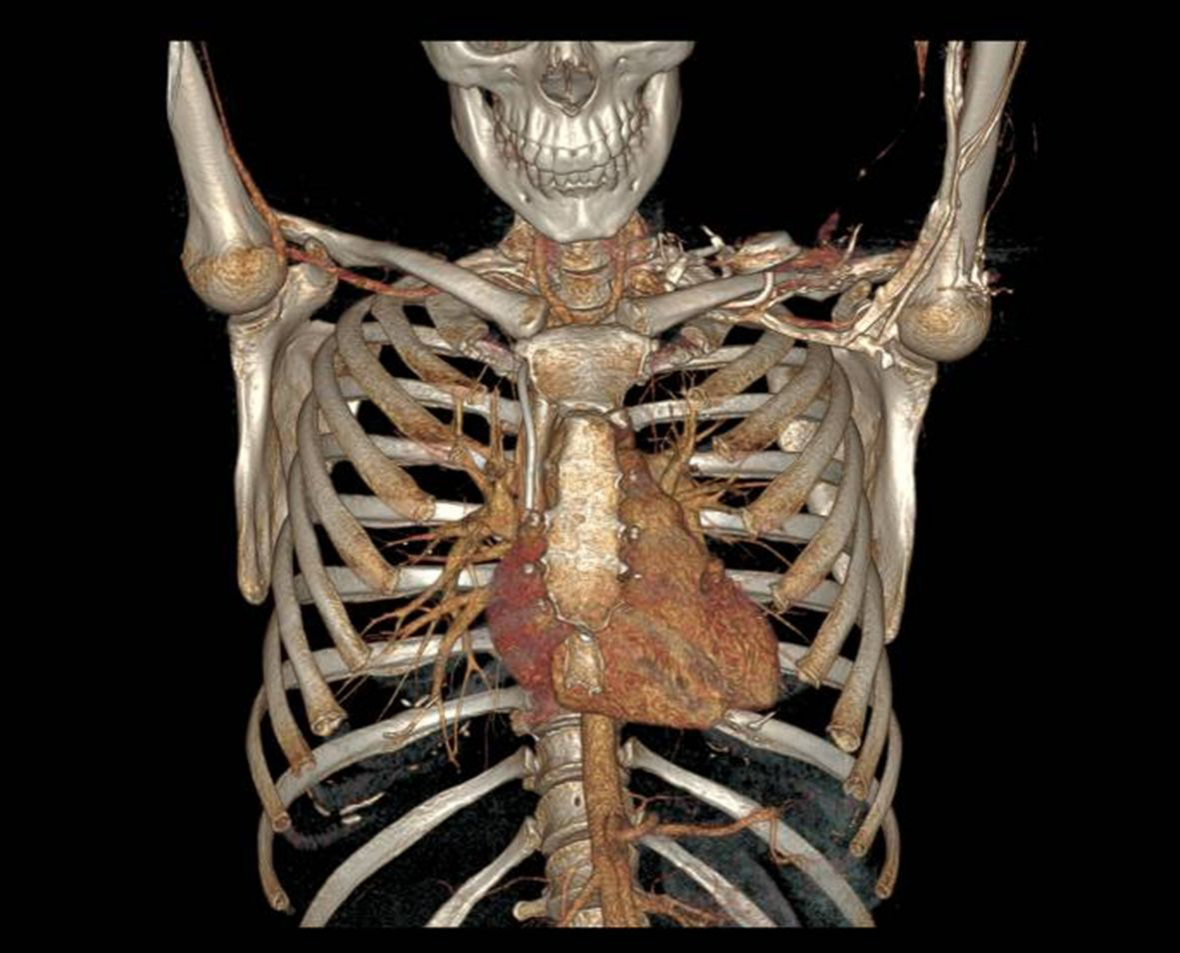
Three-dimensional computed tomography reconstruction also showing the catheter positioned in the right internal thoracic vein.

Two days later, our vascular surgery team was called because of catheter malfunction, with difficulty and pain during administration of medication. Her chest radiograph was normal, but due to persistent malfunction and pain, a chest computed tomography was performed. It showed malpositioning of the catheter, with the distal portion inserted into the right internal thoracic vein (Figure 2 and Figure 3).

The patient was taken to the operating room once more and the catheter was exchanged without any adverse events. Contrast was injected through the catheter, confirming flow into the superior vena cava. The patient recovered uneventfully from the procedure, and was discharged home to proceed with ambulatory chemotherapy. The patient gave consent for publication of this case report.

## DISCUSSION

Central venous catheters are widely used in many clinical settings, such as intensive care, oncology, anesthesia, emergency medicine and others, to deliver blood products, fluids, parenteral nutrition, chemotherapy and other drugs and for monitoring central venous pressure, pulmonary artery catheterization, transvenous cardiac pacemaker installation, and hemodialysis.^1^^,^ 2 Despite their great utility, these devices can also lead to early and late complications, some related to the insertion procedure and others related to the presence of the catheter itself. Acute complications include hematoma, pneumothorax, inadvertent arterial puncture or cannulation, failure to cannulate the target vessel, hemothorax, false path, arrhythmia, and cardiac tamponade. Catheter malpositioning can also be included in these early complications, although it can either be detected right away in the operating room or after a delay of hours or days, depending on the clinical picture.^4^ Most common late complications include infection (bacteremia or skin), thrombosis and venous stenosis.^2^

Although malpositioning of central venous catheters into the right internal thoracic vein is a relatively rare complication, it has been reported in several papers. One tends to think that many such complications are also not reported. This particular case prompted interest in publication because it produced very well-defined images and also because it had a fairly normal anteroposterior fluoroscopic image, underlining the difficulty in recognizing this complication early enough if a high level of suspicion is not maintained. Other types of malpositioning, like the tip of a subclavian catheter entering the internal jugular vein, or a jugular catheter entering the subclavian vein, seem to be more common and rarely meet the threshold for publication. We have found a case in which the central “venous” catheter was positioned into the right internal thoracic artery, providing arterial waveforms,^5^ a case that shows that there are no limits whatsoever to the different types and means of catheter malpositioning. Another curious case was an intraoperative finding of a central venous catheter inside the left internal thoracic vein during open coronary bypass grafting surgery.^6^

Inadvertent insertion of a venous catheter into the right internal thoracic vein can be quite easily overlooked during insertion, due to the apparent normal positioning in anteroposterior fluoroscopic view.^7^ The right internal thoracic vein is a tributary of the right innominate vein.^4^ It travels adjacent to the right internal thoracic artery on the posterior face of the anterior chest wall. This path means that the right internal thoracic vein overlies the superior vena cava in anteroposterior projections, so it looks normal in radiographs and fluoroscopy. On the other hand, this kind of malpositioning is easily recognized if lateral or oblique views are used.^7^ The patient in this report didn’t have lateral or oblique imaging, because the anteroposterior in-room fluoroscopy was normal. The vascular surgical team opted for computed tomography instead of a lateral chest radiograph mainly because this complication did not occur to them and to search for other possible causes of thoracic pain. Notwithstanding, computed tomography scanning can show more precisely whether or not the catheter is in an intravascular position in case of malposition, when compared to lateral chest radiography.^1^

During the insertion procedure, movement of the catheter tip should be assessed through fluoroscopy. When tip is in the correct place, it moves with changes in venous flow. On the other hand, if the catheter is fully “frozen” during fluoroscopy, care should be taken to confirm adequate positioning through oblique or lateral views, because the “frozen tip” is a warning sign of malpositioning in small veins (such as the internal thoracic vein) or in the extravascular space. Excessive movement of the catheter, resembling the cardiac cycle, may suggest malpositioning into the right atrium or even into the right ventricle. In all cases, besides oblique and lateral fluoroscopic views, intravenous iodinated contrast can also be injected through the catheter lumen, greatly helping to elucidate whether positioning is adequate or not. Since the ostium of the right thoracic vein is located in the lateral (right) wall of the innominate vein, a particularly high level of suspicion should be adopted when inserting central catheters through left subclavian and internal jugular veins, since the catheter tip often touches the right innominate vein or superior vena cava walls.

After central catheter insertion, a same-day delayed chest radiograph should always be taken to detect the presence of fresh pleural effusion or other radiographic signs, and the patient must be closely monitored for the appearance of new-onset signs and symptoms.^8^ In the case of right internal thoracic vein insertion, pain may be retrosternal, radiating to the back, during infusion of hypertonic fluids.^2^^,^ 9

In conclusion, central venous catheter malpositioning into the right internal thoracic artery is a relatively rare complication, but a high level of suspicion must be maintained, and lateral and oblique fluoroscopic views should preferably be added to supplement traditional anteroposterior imaging. If a patient develops new symptoms or signs after catheter insertion, malpositioning must be actively sought, even in the absence of pleural collections or signs of active bleeding.
